# The quality of information provided by the most popular dementia videos on TikTok

**DOI:** 10.3389/fpubh.2023.1266415

**Published:** 2023-11-28

**Authors:** Stevo Lukić, Jovana Petrović

**Affiliations:** ^1^Neurology Department, Faculty of Medicine, University of Niš, Niš, Serbia; ^2^Clinic of Neurology, University Clinical Centre Niš, Niš, Serbia; ^3^Clinic of Psychiatry, University Clinical Centre Niš, Niš, Serbia

**Keywords:** dementia, online health information, video quality, social media, TikTok, credibility, reliability

## Abstract

**Summary of background:**

Dementia is among the leading causes of death and disability worldwide, having a major impact not only on the affected person but also on all of society. The Internet is a popular and growing source of health-related information for patients, family members, carriers, and physicians. TikTok, one of the most popular social media platforms, is an important source for knowledge access and adoption. However, the quality of health information on TikTok has not been sufficiently studied.

**Objective:**

To evaluate the quality of the information provided in the most popular videos on dementia shared on TikTok.

**Study design:**

A cross-sectional study.

**Methods:**

The top 100 most popular videos on TikTok obtained by searching the hashtag “dementia” were included in the study and grouped based on their source and content. The popularity of the videos was estimated based on the numbers of likes, comments, and shares. The quality of health-related information was evaluated using the DISCERN score and the Global Quality Score (GQS).

**Results:**

Videos had a median duration of 33.29 s; the median number of likes was 635,100, with a total of 93,698,200 likes, 903,859 comments, and 5,310,912 shares. The source (uploader) of 65% of the videos was family members, while only 4% were uploaded by doctors. The content was lifestyle-related in 62% of the videos, while 12% of the videos were for fun. Videos had a median DISCERN score of 22.5 (IQR 20–27) and a median GQS of 2 (IQR 1–3). The videos uploaded by doctors had the highest quality scores and the lowest popularity.

**Conclusion:**

The most popular dementia videos on TikTok are mostly shared by family members and are of poor quality. Given the major public health issues associated with dementia, experts must provide appropriate and active assistance to patients in interpreting the information identified.

## 1 Introduction

Dementia is among the leading causes of death and disability worldwide, having a major impact not only on the affected person but also on all of society. The burden of dementia, estimated by years lived with disability, is one of the highest among all non-communicable diseases (1). Since the population of older adults is growing and the prevalence of dementia increases with age, it is logical to assume that the number of dementia cases will also increase. The World Health Organization (WHO) predicts that the prevalence of dementia will almost double every 20 years, resulting in a worldwide prevalence of 131.5 million by 2050 (2). It is anticipated that by 2030, the global cost of caring for people with dementia will have climbed to US$2 trillion, a figure that might impair global social and economic growth and overwhelm health and social services (3).

The worldwide action plan by the WHO regarding the public health response to dementia specifies the domain of public health as a priority action area, increasing dementia awareness and friendliness as well as support for dementia carers (2). Technological advancements may help to sustain cognitive function (4) or redefine effective aging at home for people with dementia by granting them continued autonomy and independence while also relieving relatives and caregivers (5). Social media platforms have the potential to make significant contributions to health communication and promotion as well as the potential to drive greater engagement with dementia research (6, 7).

The significant expansion of web-based medical information has drastically altered how consumers access health information. Before visiting a doctor, an increasing number of patients seek information online (8). With the rise in social networking platforms, people are increasingly using videos to gather medical information. However, the quality of health-related video clips on social media is unsatisfactory, and the rate of health misinformation is high (9, 10). Although health material has been widely investigated on video sites such as YouTube, studies examining online video platforms such as TikTok are rare (11).

TikTok is a short-form video hosting service that has gained global popularity since its launch, gathering over 1 billion monthly global users and growing its user base faster than any other social media platform (12). Therefore, TikTok has become another medium for sharing and seeking information globally (11). Previous studies have investigated the quality of TikTok videos regarding diabetes (13), COVID-19 (14), chronic obstructive pulmonary disease (15), and gallstone disease (16). However, the dementia material on TikTok has not yet been assessed.

With the growth of social media and Internet use, information can spread more rapidly. This can help users to acquire information more quickly but can also amplify dangerous messages. The term *infodemic* refers to a large increase in the volume of information associated with a specific topic, which can occur exponentially in a short period of time due to a specific incident, such as the COVID-19 pandemic. In this situation, misinformation and allegations appear on the scene, along with manipulation of information with uncertain intentions. This phenomenon is intensified through social networks, spreading rapidly and over long distances, like a virus (17). Accordingly, the WHO suggests risk- and evidence-based analysis and approaches to manage infodemics and reduce their impact on health behaviors (18).

On this basis, the aim of this study was to evaluate the quality of the most popular dementia videos on social media platforms, such as TikTok.

## 2 Methods

### 2.1 Data collection

We performed a cross-sectional analysis of TikTok dementia videos during June and July of 2023. This social media platform was investigated using an Android application (version 30.2.3) with a new research account. The authors independently engaged in data collection on the same day, with different mobile devices, using a single search with the hashtag “dementia”. We examined all video categories with no time limit (all dates posted), and videos were sorted by like count. At the time of data collection, each video was given a unique ID (identifying its order in the rating) and linked to its associated data in a master database (MS Access), with a screenshot of the video for verification. The target sample size was set to the first 100 unique videos. Data were collected for 108 videos, while 8 videos were excluded from further analysis based on the exclusion criteria of non-English language (*n* = 5) or animal content (*n* = 3).

For each video analyzed, we collected generic data (username, title, upload date, days since upload, source (uploader), content, and video duration) and engagement metadata (number of likes, comments, and shares). During the process of data collection and analysis, the investigator did not engage in any interactions within the application (e.g., posting comments, likes, or reactions, or messaging).

### 2.2 Classification of videos

The method of classification of videos was drawn from previous analyses of health-related topics on TikTok (13–16). The content of videos was classified as follows: (1) therapy suggestion, (2) disease description, (3) lifestyle, (4) news, (5) fun, and (6) other (unclassified). Video sources were categorized as follows: (1) doctors, (2) patients, (3) family members, (4) other medical staff, (5) news agencies (e.g., network media, newspaper, TV station, or radio station), (6) organizations (e.g., hospitals, universities, research groups, and health authorities), and (7) other (unclassified).

### 2.3 Quality assessment

The quality of the information in videos was assessed using the DISCERN instrument and the Global Quality Score (GQS).

The overall quality was assessed using the GQS, a five-point scale ranging from 1 to 5. A score of 1 represents poor quality, 2 indicates generally poor quality, 3 signifies moderate quality, 4 denotes good quality, and a maximum score of 5 reflects excellent quality (19).

The DISCERN instrument was developed to judge the quality of health information on treatment choices (20). Treatment refers to a course of action taken to address a health problem or illness, which may include self-care. Treatment options relate to the various possibilities for dealing with a health problem and include both treatments and no treatment, i.e., not taking any direct action or employing any type of treatment. The instrument is made up of 15 main questions and an overall quality assessment. Each of the 15 key questions reflects a distinct quality criterion, i.e., an important feature or standard that is a principal component of high-quality information. The overall DISCERN scores ranged from 16 to 80 and were labeled as very poor, poor, fair, good, or excellent, according to the number of points (21, 22).

The assessment and rating were performed by two reviewers independently. Reviewer 1 was a consultant neurologist, and reviewer 2 was a psychiatry resident. Potential differences between reviewers were resolved using the modified Quaker-based consensus model, which can be effectively applied in any consensus decision-making process (23). The uncertainties or disagreements most frequently raised related to the judgment of DISCERN tool items, which were originally designed for printed material. Final scores were determined by consensus.

### 2.4 Statistical analyses

Descriptive statistics are presented in the form median, interquartile range (IQR), or as a percentage, as appropriate. Differences between groups were tested using the Kruskal–Wallis test. Spearman's rank correlation coefficients, adjusted with the Bonferroni method to account for multiple comparisons, were employed to assess the relationships between variables. A significance threshold of *p* = 0.05 was applied to determine statistical significance. Statistical analysis was performed using STATA software package (StataCorp LP, USA).

### 2.5 Ethical considerations

This study used no clinical data, human specimens, or laboratory animals. All the data used in this study came from publicly available TikTok videos, and none of the data involved personal privacy concerns. In addition, the authors did not participate in any interaction, and therefore, no ethics approval was required.

## 3 Results

### 3.1 Features of dementia videos

The 100 TikTok dementia videos analyzed had a median duration of 42 s. They had received a total of 93,698,200 likes, 903,859 comments, and 5,310,912 shares. The median numbers were 635,100 likes, 4,608.5 comments, and 27,400 shares. The median number of days since upload was 176 (IQR: 101.5–284.5) at the time of data collection (Table 1).

**Table 1:** Features of the top 100 TikTok dementia videos by source.

**Video source**	**Likes**	**Comments**	**Shares**	**Days since upload**	**Duration (seconds)**
**(*****n*** = **100)**	**Median (IQR)**	**Median (IQR)**	**Median (IQR)**	**Median (IQR)**	**Median (IQR)**
Doctors (*n* = 4)	581,150 (432,950–656,450)	6,307.5 (3,584.5–7,955.5)	74,900 (36,985.5–97,650)	271.5 (158.5–279.5)	162 (122–175)
Patients (*n* = 2)	1,108,950 (417,900–1,800,000)	12,170.5 (9,241–15,100)	176,550 (141,600–211,500)	88 (60–116)	9.5 (6–13)
Family members (*n* = 65)	620,600 (416,800–963,200)	5,251 (1,942–10,500)	19,800 (6,285–40,900)	312 (131–351)	18.5 (15–40)
Other medical staff (*n* = 14)	840,900 (444,900–1,800,000)	2,530 (897–18,300)	13,450 (1,921–105,200)	208.9 (216.9)	37 (35.16)
News agency (portals) (*n* = 3)	772,100 (567,800–776,400)	3,145 (1,011–11,100)	34,900 (12,200–52,400)	97 (53–211)	46 (21–48)
Organizations (*n* = 5)	719,000 (449,200–782,900)	2,625 (2,285–3,079)	46,700 (40,100–54,800)	200 (172–341)	35 (22–45)
Other (*n* = 7)	630,500 (483,200–2,000,000)	4,361 (2,904–13,300)	50,900 (41,700–71,400)	114 (50–272)	21 (8–49)
Total	635,100 (433,400–1,039,950)	4,608.5 (1,849.5–10,600)	27,400 (6,453–6,453)	176 (101.5–284.5)	42 (18–78.5)

IQR, interquartile range.

Regarding video sources, 65% (65 of 100) of the videos were posted by family members, while only 4% (4 of 100) were posted by doctors. Other video sources were patients at 2% (2 of 100), other medical staff at 14% (14 of 100), news agencies at 3% (3 of 100), organizations at 5% (5 of 100), and other (unclassified) sources at 7% (7 of 100).

Regarding the content, lifestyle videos were the most dominant, accounting for 62% (62 of 100) of all the videos. The proportions of other types of content were 22% (22 of 100) for fun, 12% (12 of 100) for disease description, and 2% (2 of 10) for news, while therapy suggestions and other types content covered 1% (1 of 100) each (Table 2).

**Table 2:** Features of the top 100 TikTok dementia videos by type of content.

**Video content**	**Likes**	**Comments**	**Shares**	**Days since upload**	**Duration (seconds)**
**(*****n*** = **100)**	**Median (IQR)**	**Median (IQR)**	**Median (IQR)**	**Median (IQR)**	**Median (IQR)**
Therapy suggestions (*n* = 1)	776,400	3,145	52,400	97	48
Disease description (*n* = 12)	708,550 (560,450–1,125,800)	5,131 (2,171–8,867.5)	35,650 (2,431–82,500)	236 (72–298)	51.5 (34–87.5)
Lifestyle (*n* = 62)	612,050 (407,400–963,200)	4,319.5 (1,819–10,500)	16,000 (5,499–46,000)	176 (129–272)	52.5 (21–83)
News (*n* = 2)	557,650 (547,500–567,800)	5,106.5 (1,011–9,202)	45,600 (12,200–79,000)	162.5 (53–272)	108 (46–170)
Fun (*n* = 22)	674,750 (483,200–1,800,000)	4,589.5 (1,942–11,100)	52,550 (25,600–123,700)	172.5 (96–313)	14.5 (8–26)
Other (*n* = 1)	654,700	11,100	22,000	67	164

IQR, interquartile range.

### 3.2 Video quality assessments

**Figure 1:**
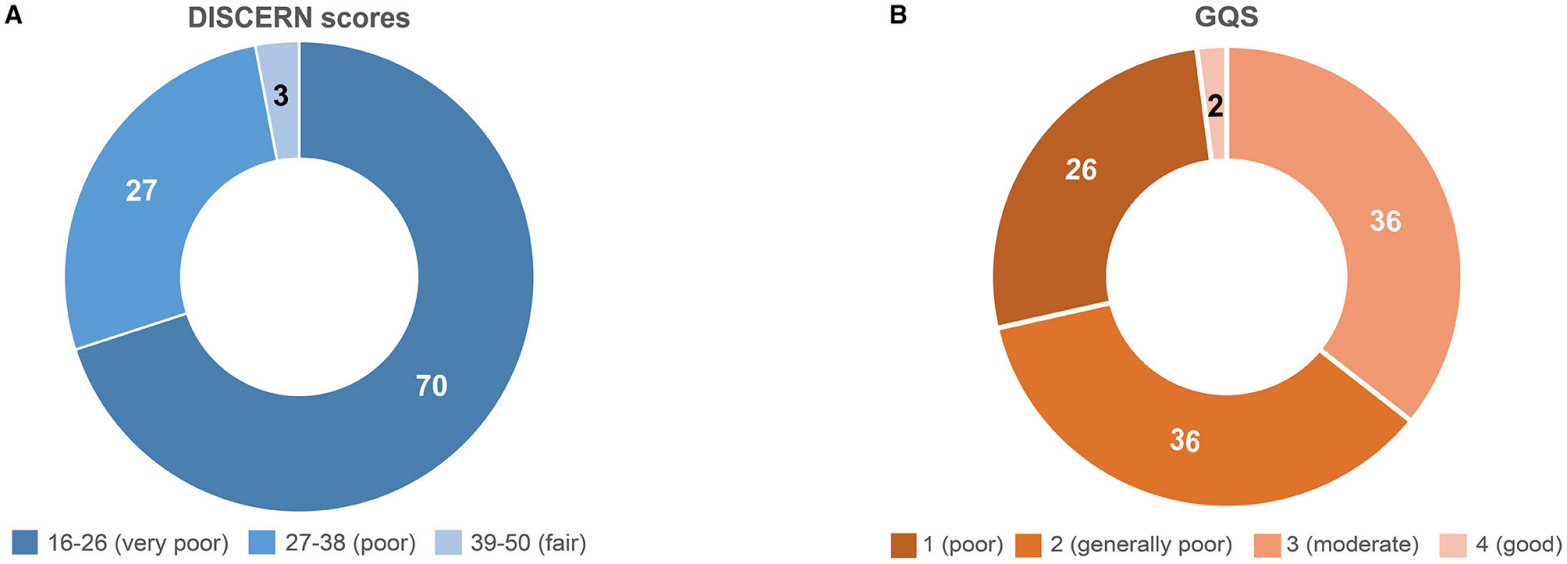
DISCERN and Global Quality Scores of the top 100 most popular dementia videos on TikTok. **(A)** DISCERN scores (5 levels); **(B)** Global Quality Scores (GQS). Colors represent the quality scores of videos; darker colors correspond to lower quality. The numbers in the figures represent the percentage of videos in each category.

According to the DISCERN and GQS instruments, the quality of dementia-related videos on TikTok was very low. The median DISCERN and GQS scores for all 100 top videos were 22.5 (IQR 20–27) and 2 (IQR 1–3), respectively. Most videos were very poor or poor quality according to their DISCERN score and generally poor or moderate according to their GQS score (Table 3; Figure 1).

**Table 3:** DISCERN and Global Quality Scores for the top 100 TikTok dementia videos.

**Score**	**Percentage (%)**
**DISCERN**	
16–26 (very poor)	70
27–38 (poor)	27
39–50 (fair)	3
51–62 (good)	/
63–80 (excellent)	/
**GQS score**	
1 (poor)	26
2 (generally poor)	36
3 (moderate)	36
4 (good)	2
5 (excellent)	/

GQS, Global Quality Score.

**Figure 2:**
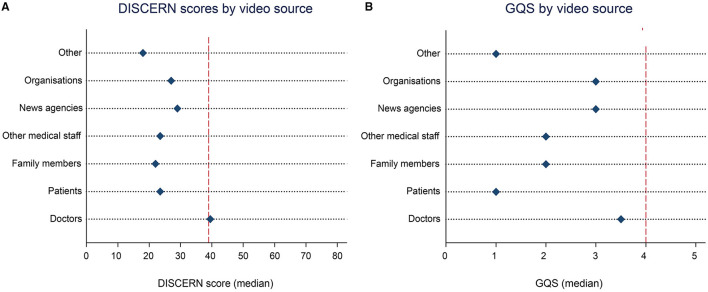
Quality assessments of the top 100 TikTok dementia videos by source. **(A)** Median DISCERN score by video source. Reference line: DISCERN score = 39 (lower limit for “fair” quality). **(B)** Median GQS by video source. Reference line: GQS = 4 (lower limit for “good” quality). GQS, Global Quality Score.

The quality of videos was found to be significantly related to video source and content. Only videos uploaded by doctors approached the reference line for “fair” quality according to DISCERN score and “good” quality on the GQS scale (Figure 2). The quality of doctors' videos as measured by as DISCERN score (median 39.5, IQR 34.5–40.5) was significantly better than that of other videos (Kruskal–Wallis test, chi-squared: 19.761, df = 6, *p* < 0.01). In addition, the quality of doctors' videos as measured by GQS (median 3.5, IQR 3–4) was significantly different from that of other videos (Kruskal–Wallis test, chi-squared: 19.578, df = 6, *p* < 0.01).

**Figure 3:**
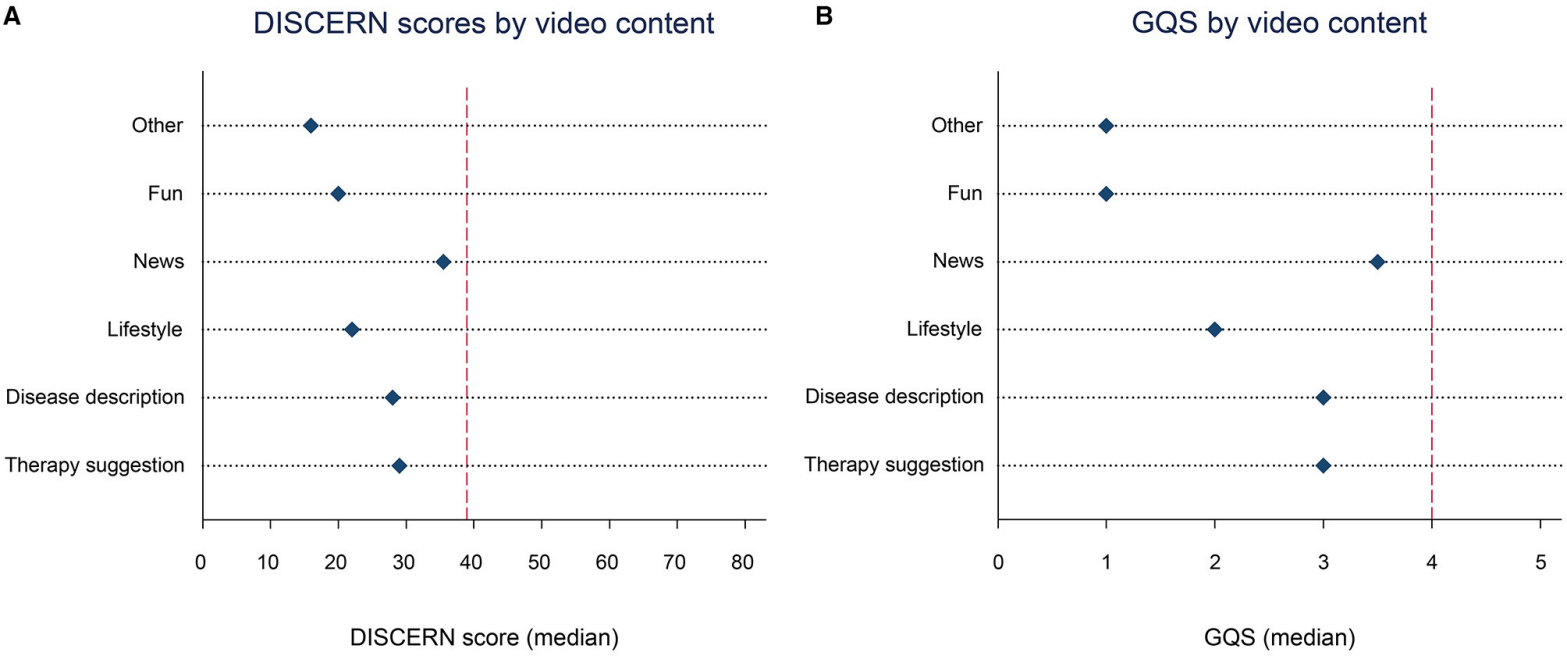
Quality assessments of the top 100 TikTok dementia videos by content. **(A)** Median DISCERN score by video content. Reference line: DISCERN score = 39 (lower limit for “fair” quality). **(B)** Median GQS by video content. Reference line: GQS = 4 (lower limit for “good” quality). GQS, Global Quality Score.

Regarding the video content, most videos were of poor or very poor quality. Videos with news content (median DISCERN score: 35.5, IQR 31–40; median GQS: 3.5, IQR 3–4) were of significantly higher quality in comparison to other videos (Kruskal–Wallis test, chi-squared: 26.352, df = 5, *p* < 0.01 for DISCERN; chi-squared: 24.342, df = 5, *p* < 0.01 for GQS) (Figure 3).

Although patients' videos received the most likes, comments, and shares, they were of very poor quality according to the instruments used.

The correlation analysis revealed connections between the following parameters: likes and comments (rho = 0.48, *p* < 0.01); likes and shares (rho = 0.51, *p* < 0.01); comments and shares (rho = 0.54, *p* < 0.01); comments and duration (rho = −0.32, *p* < 0.05); and shares and days since upload (−0.3382, *p* < 0.05; Table 4).

**Table 4:** Results of the correlation analysis between video-related variables.

	**Likes**	**Comments**	**Shares**	**Days**	**Duration**
Likes	-	-	-	-	-
Comments	0.4812^**^	-	-	-	-
Shares	0.5094^**^	0.5379^**^	-	-	-
Days	0.0087	−0.0202	−0.3382^**^	-	-
Duration	0.0087	0.3201^*^	0.1045	−0.0683	-

The results represent Spearman's rank correlation coefficients with Bonferroni adjustment for calculation of the significance level.

^*^p < 0.05.

^**^p < 0.01.

With the exception of video duration, which showed positive correlations with DISCERN and GQS at rho = 0.3881 and rho = 0.4208, respectively (*p* < 0.05 for both), we observed no significant associations between video quality scores and the other video-related variables (Table 5).

**Table 5:** Results of analysis of the correlation between video quality scores and video-related variables.

	**DISCERN**	**GQS**
Likes	0.1187	−0.0640
Comments	−0.0165	−0.0541
Shares	−0.0744	−0.1917
Days	0.0872	0.0452
Duration	0.3881^**^	0.4208^**^

The results represent Spearman's rank correlation coefficients with Bonferroni adjustment for calculation of the significance level.

^**^p < 0.01.

GQS, Global Quality Score.

## 4 Discussion

The main finding of this study is that the overall quality of the information in dementia-related videos on TikTok is very low and differs significantly depending on the source. The most popular dementia videos are mainly provided by family members and are of low quality. The infrequent videos uploaded by doctors had the highest quality scores and the lowest popularity.

We discovered positive correlations between likes, comments, and shares, showing that popular videos were more likely to gather comments and to be shared. The number of comments was also found to be positively associated with the number of shares, implying that videos with more comments are more likely to be shared. The number of days since upload was not correlated with number of likes and comments, but was negatively correlated with number of shares, showing that there was no time-dependent influence on popularity. It is notable that there was no link between video length and number of likes and shares. With the exception of a link between duration and quality, implying that longer videos were of higher quality, we discovered no associations between video parameters and quality scores.

Overall, this study found that the most popular videos about dementia on TikTok are of the lowest academic and educational quality. These findings suggest that TikTok users are unable to distinguish between high-quality and low-quality videos. The results may be connected to the characteristics of TikTok users. Since TikTok is primarily a lifestyle activity platform, its users prefer entertaining videos, and videos with pleasant graphics are more attractive. Videos with better credibility are not popular, most likely because professional content is serious or even monotonous, making it difficult for such videos to gain popularity.

Some studies suggest that users may be more likely to seek out video information that differs from standard medical procedures (24). Content that is unconventional and differs from conventional medical recommendations may be more attractive to users and thus gain more views and likes. Educational videos may not be as exciting, which makes them less interesting to non-professionals.

The primary limitation of our study is that we analyzed only the first 100 videos obtained by searching the hashtag “dementia”. Previous studies have found that most users do not read more than one or two pages of online search results (25), and that it is unlikely for users to read beyond the first 50 search engine results (26, 27). Therefore, we evaluated the top 100 videos obtained via this search.

Another possible limitation is that videos were sorted by number of likes. We chose this method because the primary goal of the study was to assess the quality of the most popular videos that may have the largest public impact. In addition, we used this method to reduce potential location effects, since the default settings of the application may differ depending on the user's Internet Protocol address or other unknown conditions. Similarly, the search was performed at a single timepoint without engagement in any interaction (e.g., likes, comments, reactions, or messages) to minimize adjustment or recommendations by social networking algorithms (28) and to enable better reproducibility of the results.

In addition, we did not analyze the characteristics of music the associated with the videos. Background music does not considerably improve the popularity of videos, according to a previous study (29). Therefore, the evaluation of the technical quality of the video image or the included music are beyond the scope of this study.

Furthermore, although we performed an analysis of engagement data (likes, comments, and shares), we did not conduct a deeper investigation into the content of the comments. Some viewers may make comments out of displeasure, and these unfavorable reviews may boost the number of comments. However, we identified strong positive correlations among the numbers of comments, likes, and shares, which corresponds to positive rather than negative popularity. Further studies should focus on analysis of the association between positive and negative comments and video quality.

Thus, despite potential limitations, we feel that this study established an objective framework for assessment of the quality of the most popular dementia videos on TikTok.

Several studies have been conducted on health-related content on TikTok, and these have used different instruments (13–16, 30–33). Some studies have evaluated videos only based on engagement data (14), using minimal tools (30, 31), or according to clinical guidelines (33). However, we have used the most comprehensive and most frequently employed instruments: DISCERN and the GQS (13, 15, 16, 32). In addition, previous studies have found that the JAMA benchmark criteria (34) could not accurately assess video information and were not precise enough (16). Regarding the topic, research exploring mental health content on TikTok is essentially absent from the published research literature (30). To the best of our knowledge, this is the first study to evaluate the quality and reliability of information regarding dementia on TikTok. We believe that this novel study will facilitate further investigation and serve as a reference comparator for research examining dementia content on this social media platform. The videos analyzed in our study had more than 93 million likes, suggesting that there is substantial interest in content providing information on dementia on TikTok. This indicates great public interest and is a potential avenue for promising interventions. Specifically, increasing the number of videos uploaded by doctors or academic groups and ensuring that content remains engaging while maintaining a professional standard could enhance audience engagement and contribute to the wider dissemination of accurate medical knowledge.

The clinical characteristics of dementia, reflected by changes in personality and mental capacity, frequently necessitate ongoing care, which can be extremely taxing physically and emotionally for the family members or professional caregivers who handle most of this care. Additionally, it is common for family members of dementia patients to not receive enough information or counsel on the disease, including information on the legal, financial, diagnostic, and treatment-related aspects of the disease (35). Furthermore, the stress of providing dementia care is linked to a wide range of physical and mental health conditions and negatively affects the quality of life of both care recipients and carers. Interventions for dementia carers have mostly concentrated on education and skill development with the aim of reducing feelings of stress and workload (36). Social media platforms have the potential to significantly contribute to health communication and to improve public health outcomes by increasing dementia awareness through a public campaign to promote a dementia-inclusive society (2).

## 5 Conclusion

This study shows that less accurate and less reliable dementia videos are more favored by TikTok users. However, due to the growing popularity of this platform and the major burden imposed by dementia, the potential of public health promotion via this platform cannot be overlooked. More videos created by health professionals and refined via a serious review process may increase health knowledge and public awareness of dementia, as well as support for dementia caregivers.

## Data availability statement

The raw data supporting the conclusions of this article will be made available by the authors, without undue reservation.

## Author contributions

SL: Conceptualization, Formal analysis, Methodology, Software, Supervision, Writing – original draft, Writing – review & editing. JP: Data curation, Methodology, Writing – original draft, Writing – review & editing.

## References

[1] Prince M Albanese E Guerchet M Prina M. World Alzheimer Report 2014. Dementia and Risk Reduction: An Analysis of Protective and Modifiable Factors. London: Alzheimer's Disease International (2014). p. 104.

[2] Global Action Plan on the Public Health Response to Dementia 2017-2025. Geneva: World Health Organization (2017). p. 27.

[3] Prince M Wimo A Guerchet M Ali GC Wu Y Prina M. World Alzheimer Report 2015. The Global Impact of Dementia: An Analysis of Prevalence, Incidence, Cost and Trends. London: Alzheimer's Disease International (2015). p. 87.

[4] Cho G Betensky RA Chang VW. Internet usage and the prospective risk of dementia: a population-based cohort study. J Am Geriatr Soc. (2023) 71:2419–29. 10.1111/jgs.18394 37132331 PMC13105409

[5] Shu S Woo BK. Use of technology and social media in dementia care: current and future directions. World J Psychiatry. (2021) 11:109–23. 10.5498/wjp.v11.i4.109 33889536 PMC8040150

[6] Hrincu V An Z Joseph K Jiang YF Robillard JM. Dementia research on Facebook and Twitter: current practice and challenges. J Alzheimers Dis. (2022) 90:447–59. 10.3233/JAD-220525 36155513 PMC9697056

[7] Martínez-Pérez B de la Torre-Díez I Bargiela-Flórez B López-Coronado M Rodrigues JJ. Content analysis of neurodegenerative and mental diseases social groups. Health Inform J. (2015) 21:267–83. 10.1177/1460458214525615 24698768

[8] McMullan RD Berle D Arnáez S Starcevic V. The relationships between health anxiety, online health information seeking, and cyberchondria: systematic review and meta-analysis. J Affect Disord. (2019) 245:270–8. 10.1016/j.jad.2018.11.037 30419526

[9] Khullar D. Social media and medical misinformation: confronting new variants of an old problem. JAMA. (2022) 328:1393–4. 10.1001/jama.2022.17191 36149664

[10] Suarez-Lledo V Alvarez-Galvez J. Prevalence of health misinformation on social media: systematic review. J Med Internet Res. (2021) 23:e17187. 10.2196/17187 33470931 PMC7857950

[11] Eghtesadi M Florea A. Facebook, Instagram, Reddit and TikTok: a proposal for health authorities to integrate popular social media platforms in contingency planning amid a global pandemic outbreak. Can J Public Health. (2020) 111:389–91. 10.17269/s41997-020-00343-0 32519085 PMC7282468

[12] Zenone M Ow N Barbic S. TikTok and public health: a proposed research agenda. BMJ Glob Health. (2021) 6:e007648. 10.1136/bmjgh-2021-007648 34819326 PMC8614045

[13] Kong W Song S Zhao YC Zhu Q Sha L. TikTok as a health information source: assessment of the quality of information in diabetes-related videos. J Med Internet Res. (2021) 23:e30409. 10.2196/30409 34468327 PMC8444042

[14] Ostrovsky AM Chen JR. TikTok and its role in Covid-19 information propagation. J Adolesc Health. (2020) 67:730. 10.1016/j.jadohealth.2020.07.039 32873499 PMC7455791

[15] Song S Xue X Zhao YC Li J Zhu Q Zhao M. Short-video apps as a health information source for chronic obstructive pulmonary disease: information quality assessment of TikTok videos. J Med Internet Res. (2021) 23:e28318. 10.2196/28318 34931996 PMC8726035

[16] Sun F Zheng S Wu J. Quality of information in gallstone disease videos on TikTok: cross-sectional study. J Med Internet Res. (2023) 25:e39162. 10.2196/39162 36753307 PMC9947761

[17] Zarocostas J. How to fight an infodemic. Lancet. (2020) 395:676. 10.1016/S0140-6736(20)30461-X 32113495 PMC7133615

[18] WHO/UNICEF. How to Build an Infodemic Insights Report in 6 Steps. San Francisco, CA: World Health Organization and the United Nations Children's Fund (UNICEF) (2023).

[19] Bernard A Langille M Hughes S Rose C Leddin D Veldhuyzen van Zanten S. A systematic review of patient inflammatory bowel disease information resources on the World Wide Web. Am J Gastroenterol. (2007) 102:2070–7. 10.1111/j.1572-0241.2007.01325.x 17511753

[20] Charnock D Shepperd S Needham G Gann R. DISCERN: an instrument for judging the quality of written consumer health information on treatment choices. J Epidemiol Community Health. (1999) 53:105–11. 10.1136/jech.53.2.105 10396471 PMC1756830

[21] Modiri O Guha D Alotaibi NM Ibrahim GM Lipsman N Fallah A. Readability and quality of wikipedia pages on neurosurgical topics. Clin Neurol Neurosurg. (2018) 166:66–70. 10.1016/j.clineuro.2018.01.021 29408776

[22] Yacob M Lotfi S Tang S Jetty P. Wikipedia in vascular surgery medical education: comparative study. JMIR Med Educ. (2020) 6:e18076. 10.2196/18076 32417754 PMC7334757

[23] Dressler L. Consensus Through Conversation. San Francisco, CA: Berrett-Koehler Publishers, Inc. (2006).

[24] Lenczowski E Dahiya M. Psoriasis and the Digital Landscape: YouTube as an information source for patients and medical professionals. J Clin Aesthet Dermatol. (2018) 11:36–8. 29606999 PMC5868783

[25] Morahan-Martin JM. How internet users find, evaluate, and use online health information: a cross-cultural review. Cyberpsychol Behav. (2004) 7:497–510. 10.1089/cpb.2004.7.497 15667044

[26] Hanif F Abayasekara K Willcocks L Jolly EC Jamieson NV Praseedom RK. The quality of information about kidney transplantation on the World Wide Web. Clin Transplant. (2007) 21:371–6. 10.1111/j.1399-0012.2006.00652.x 17488387

[27] Chestnutt IG. The nature and quality of periodontal related patient information on the world-wide web. Br Dent J. (2002) 193:657–9. 10.1038/sj.bdj.4801653 12607624

[28] Anderson KE. Getting acquainted with social networks and apps: it is time to talk about TikTok. Library Hi Tech News. (2020) 37:7–12. 10.1108/LHTN-01-2020-0001

[29] Mueller SM Hongler VNS Jungo P Cajacob L Schwegler S Steveling EH. Fiction, falsehoods, and few facts: cross-sectional study on the content-related quality of atopic eczema-related videos on YouTube. J Med Internet Res. (2020) 22:e15599. 10.2196/15599 32329744 PMC7210495

[30] Basch CH Donelle L Fera J Jaime C. Deconstructing TikTok videos on mental health: Cross-sectional, descriptive content analysis. JMIR Form Res. (2022) 6:e38340. 10.2196/38340 35588057 PMC9164092

[31] Chasca W Nerada S Zenone M Barbic S. TikTok and #OccupationalTherapy: cross-sectional study. JMIR Form Res. (2023) 7:e45554. 10.2196/45554 37204836 PMC10238957

[32] McBriar JD Mishra A Shah HA Boockvar JA Langer DJ D'Amico RS. Neurosurgery: a cross-sectional analysis of neurosurgical content on TikTok. World Neurosurg. (2022) 17:100137. 10.1016/j.wnsx.2022.100137 36204176 PMC9529591

[33] Oulee A Ivanic M Norden A Javadi SS Wu JJ. Atopic dermatitis on TikTok™: a cross-sectional study. Clin Exp Dermatol. (2022) 47:2036–7. 10.1111/ced.15322 35796132

[34] Silberg WM Lundberg GD Musacchio RA. Assessing, controlling, and assuring the quality of medical information on the Internet: Caveant lector et viewor- Let the reader and viewer beware. JAMA. (1997) 277:1244–5. 10.1001/jama.1997.03540390074039 9103351

[35] Steiner V Pierce LL Salvador D. Information needs of family caregivers of people with dementia. Rehabil Nurs. (2016) 41:162–9. 10.1002/rnj.214 25858031

[36] Moskowitz JT Cheung EO Snowberg KE Verstaen A Merrilees J Salsman JM. Randomized controlled trial of a facilitated online positive emotion regulation intervention for dementia caregivers. Health Psychol. (2019) 38:391–402. 10.1037/hea0000680 31045422 PMC6501812

